# P-1462. No All Pneumococcus Serogroups Are the Same. Correlation of Serotype and Sequence Type (ST) by Multilocus Sequence Typing (MLST) in Invasive Isolates in Children

**DOI:** 10.1093/ofid/ofaf695.1648

**Published:** 2026-01-11

**Authors:** Jose Alexander

**Affiliations:** Advent Health Central Florida, Orlando, FL

## Abstract

**Background:**

*Streptococcus pneumoniae* is a leading cause of invasive infections, particularly in children. Genotyping is crucial for epidemiological studies and vaccine formulation. Sequence Type (ST) by Multilocus Sequence Typing (MLST) through whole genome sequencing (WGS) using long-read platforms, such as Oxford Nanopore, offers an accessible and rapid in-house alternative to reference laboratories. This study evaluates the correlation between serogroups and ST from invasive *S. pneumoniae* infection.

**Methods:**

Thirteen *S. pneumoniae* isolates from invasive infections in children were analyzed by WGS at AdventHealth Orlando. Isolates were cultured on blood agar plate and DNA extracted using the ZymoBIOMICS MagBead 96 DNA Kit (Zymo Research). Library preparation was performed with the Rapid Sequencing Kit V14 (Oxford Nanopore) and sequenced at one isolate per flow-cell (Flongle, Oxford Nanopore) for 22 hours. The resulting FAST file was uploaded to the free Pasteur Institute's website for ST by MLST and serotype correlation following the website step-by-step workflow.

**Results:**

MLST and ST analysis of all thirteen isolates revealed ten unique serogroups distributed among 12 different ST. 35B (n=2), 19F (n=2), and 15BC (n=2) were the only serogroups with two isolates each. Both 35B and 19F had different sequence types, ST558 and ST156 in 35B, and ST1203 and ST251 in 19F. Both 15BC isolates were carried by the same ST7479. ST558 is a known beta-lactam resistant strain that switched from 19A to 35B. Thus, as ST156, a multidrug resistant strain switching from 9V and 19A to 35B. ST1203 19F is a globally persistent MDR clone form Taiwan19F-14, and ST251 19F is a less common MDR strain with multiple capsular switching such as 19A, 23F, 35B, 15A, allowing to escape vaccination. 15BC a known invasive serogroup has very limited information regarding its ST7479 strain.

**Conclusion:**

This study demonstrates the overlapping of serogroups and ST when tracking *S. pneumonia* and their related virulence and resistance. Correlation of serotype and ST for awareness of capsular switching as an epidemiological tool for regional prevalence, treatment approach, and vaccination plan, are the goals of this program. Only serotype testing maybe misleading, when the ST provide a broader virulence and resistance profile.

**Disclosures:**

All Authors: No reported disclosures

